# Diffusion and function of Wnt ligands

**DOI:** 10.1371/journal.pgen.1008154

**Published:** 2019-06-13

**Authors:** Richard A. Stewart, Aravinda-Bharathi Ramakrishnan, Ken M. Cadigan

**Affiliations:** Department of Molecular, Cellular and Developmental Biology, University of Michigan, Ann Arbor, Michigan, United States of America; Stanford University School of Medicine, UNITED STATES

Wnts are secreted, lipidated glycoproteins that are essential for cell–cell communication in development and tissue homeostasis throughout metazoans [1–3]. The “W” in Wnt comes from the *wingless* (*wg*) gene, which is required for proper formation of most tissues and appendages in *Drosophila* [4,5]. Immunostainings showed gradients of Wg protein emanating from Wg-producing cells in embryos and larval imaginal discs [6–9]. In the mid-1990s, elegant genetic studies in the wing imaginal discs supported the hypothesis that Wg protein could act as a diffusible morphogen by specifying different cell fates along a concentration gradient [10,11]. Although this evidence seemed compelling, caveats included perdurance from earlier Wg expression throughout the presumptive wing and the potential spread of the signal through the division of Wg-producing cells [4,12]. Still, Wg has commonly been considered a paradigm for a secreted protein that can act as a diffusible morphogen across a developing tissue [13–15].

**Fig 1:**
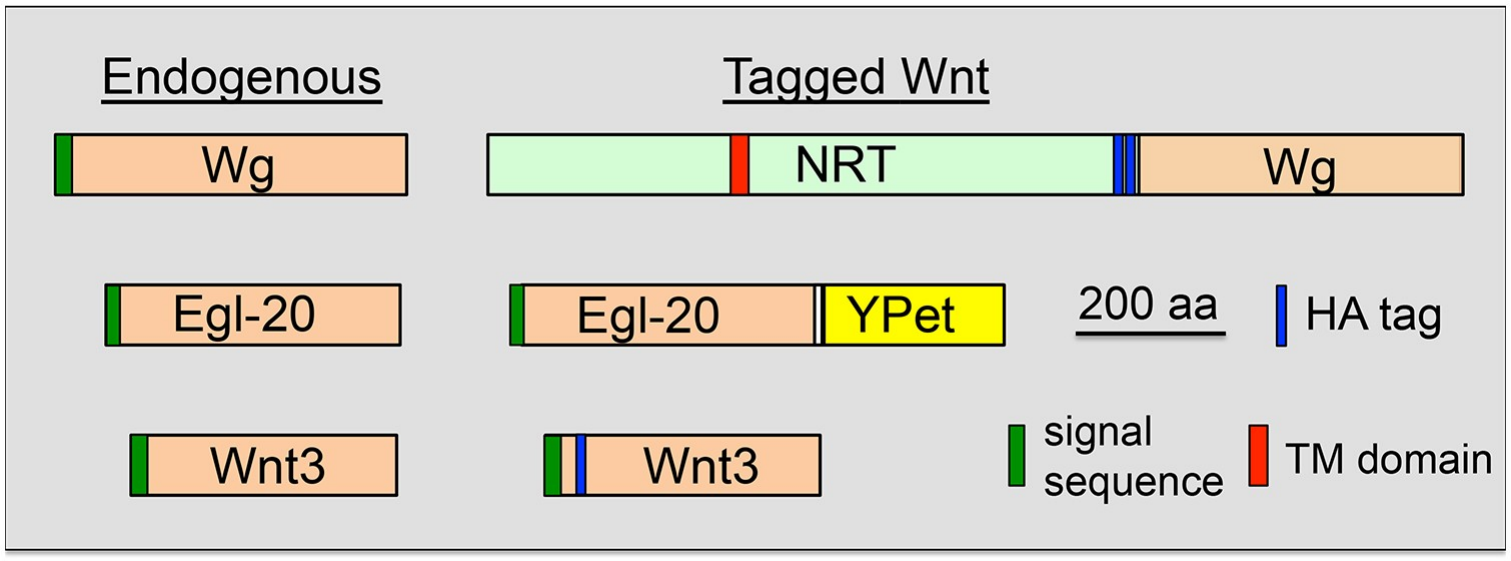
Several constructs used to study Wnt diffusion. Endogenous Wg, Egl-20, and murine Wnt3 are depicted on the left, with their N-terminal signal sequences. NRT-Wg, Egl-20-YPet, and HA-tagged Wnt3 are shown on the right. NRT is a type II TM protein, so the C-terminal portion containing Wg will be extracellular. aa, amino acid; Egl-20, egg-laying defective-20; HA, hemagglutinin; NRT, neurotactin TM domain; TM, transmembrane; Wg, wingless; YPet, a yellow fluorescent protein derived from green fluorescent protein [29].

The importance of Wg diffusion was tested by Alexandre and colleagues in 2014, when they replaced the endogenous Wg gene with a cDNA encoding a fusion of the neurotactin transmembrane domain and Wg (NRT-Wg; Fig 1). Tethering Wg to the plasma membrane of Wg-producing cells resulted in a dramatic restriction of the Wg protein gradient in late-stage wing imaginal discs [16]. NRT-Wg homozygotes, i.e., flies in which NRT-Wg is their only source of Wg, survive to adulthood with relatively mild phenotypes, such as slightly smaller wings [16]. This is surprising, as *wg* is essential for viability and patterning at embryonic and larval stages [4]. The authors suggested that perdurance of Wg target gene expression from earlier stages could provide the information necessary for proper patterning [16]. A recent preprint reports that NRT-Wg is more stable than Wg, in part because of increased expression of its receptor, frizzled 2 (Fz2) [17], which can stabilize Wg [8,17]. These nuances do not detract from the seminal conclusion of Alexandre and colleagues, i.e., that tethering of Wg to its producing cells has almost no effect on the patterning of the fly.

Although NRT-Wg flies make it to adulthood, they are sterile and developmentally delayed [16]. In addition, as shown by a new report in this issue, NRT-Wg flies have severe defects in cell fate specification, proliferation, and morphology of the adult intestine [18]. Wg is normally expressed at high levels at the midgut–hindgut boundary (MHB) of the intestine, where it activates Wg target genes at a distance from the MHB [18]. The area of Wg target gene expression is severely reduced in NRT-Wg intestines. The structure of the MHB is drastically altered, as are the longitudinal and circular muscles surrounding the intestine [18]. Epithelial patterning and proliferation are also disrupted. These defects could be phenocopied by a reduction of Wg signaling, and NRT-Wg phenotypes were not rescued by overexpression of a NRT-Wg transgene. NRT-Wg flies had decreased longevity compared with controls, which was more severe when fed a sucrose-only diet [18]. Taken together, the data support a model in which Wg spreading is essential for proper development and function of the intestine. NRT-Wg flies also have developmental defects in the embryonic ureter/Malphigian tubules [19] and neuronal specification in the visual system [20]. These results indicate that in some contexts, Wg may indeed act as a diffusible morphogen.

Debate over the morphogen properties of Wg and other Wnts has been fueled by a lack of visual evidence that Wnt proteins can spread from their sites of synthesis. Tagging Wnts with fluorescent proteins has historically been challenging, as the modification typically lowers their biological activity [1]. However, this hurdle was recently overcome for Egl-20 [21], a nematode Wnt important for specific neuroblast specification/migration and axonal guidance. CRISPR/Cas9-based tagging of Egl-20 with mNeonGreen or mYpet at its C terminus (Fig 1) resulted in a functional protein with none of the phenotypes normally associated with *egl-20* mutants [21]. Live imaging in these worms demonstrated that Egl-20 forms a gradient along the anteroposterior axis, with the strongest fluorescent signal colocalizing with source cells. Fluorescence recovery after photobleaching (FRAP) experiments revealed that the Wnt fluorescent signal is dynamic and can quickly recover, on a scale of <5 minutes, indicating that Wnt spreads rapidly. Additionally, sequestering extracellular, fluorescently labeled Wnt ligands using a nanobody resulted in cell migration defects that are consistent with *egl-20* loss of function, demonstrating a long-range function for Egl-20 [21]. This study provides direct evidence that some Wnt proteins have long-range signaling capabilities.

In contrast to *Caenorhabditis elegans* Egl-20, it appears that Wnt signaling in the crypts of the mammalian small intestine occurs over a short range [22]. In this tissue, Wnt signaling is required for the maintenance of intestine stem cells (ISCs) and proliferation of transit-amplifying cells [2,23]. Adjacent to the ISCs, Paneth cells provide one source of Wnt ligands [2]. Wnt3 is expressed in Paneth cells, and when the endogenous gene was hemagglutinin (HA) tagged (Fig 1), its expression pattern suggested that it signals in a juxtracrine manner [22]. Frizzled receptors may act as tethers for extracellular Wnt3, and the protein appears to spread from the Paneth cells via mitosis of ISCs [22]. In addition, the mesenchyme surrounding the crypts is a source of Wnt ligands. Two recent papers identify FoxL1 and PDGFα-positive subepithelial telocytes as an essential source of Wnt signals [24,25]. Transcriptional profiling revealed that telocytes near different crypt zones produce different levels of Wnt. This suggests a model in which a Wnt gradient arises from different levels of transcription in Wnt-producing mesenchyme rather than long-range transport of Wnts from a single source [24].

Perhaps it is not surprising that the range of Wnt signaling varies dramatically in different contexts. Though not discussed in this article, there is also evidence that Wnts, including Wg, can move away from sites of synthesis via membrane extensions, e.g., cytonemes [26,27], as well as exosomes, microvesicles, and lipoproteins [28]. Whether NRT-Wg protein utilizes these mechanisms remains to be explored. Moving forward, using genome editing to fluorescently tag or membrane tether more Wnts will expand our knowledge of the systems and differentiate between contexts that require long- and short-range Wnt signals.
